# The impact of transcatheter aortic valve replacement on changes of coronary computed tomography-derived fractional flow reserve

**DOI:** 10.1080/07853890.2024.2420860

**Published:** 2024-10-28

**Authors:** Feng Hu, Qianyao Lai, Jun Fang, Xi He, Chaoyang Lin, Mingming Hu, Lin Fan, Lianglong Chen

**Affiliations:** ^a^ Department of Cardiology, Fujian Medical University Union Hospital, Fujian Cardiovascular Medical Center, Fujian Institute of Coronary Artery Disease, Fujian Cardiovascular Research Center, Fuzhou, P. R. China; ^b^ School of Health, Fujian Medical University, Fuzhou, P. R. China; ^c^ Pulse Medical Technology Company, Shanghai, P. R. China

**Keywords:** Computed tomography-derived fractional flow reserve, transcatheter aortic valve replacement, plaque burden, cardiac function

## Abstract

**Background:**

The effect of transcatheter aortic valve replacement (TAVR) on changes of computed tomography-derived fractional flow reserve (CT-FFR) values was controversial. Thus, we aimed to identify the impact of TAVR on changes of CT-FFR values, plaque characteristics, and the associated clinical impact.

**Methods:**

This single-center observational study included 39 consecutive patients with severe aortic valve disease undergone TAVR between August 2019 and April 2023, whom were performed with preoperative and postoperative coronary CT angiography (CCTA). The computation of CT-FFR and plaque characteristics was performed by an independent central core laboratory.

**Results:**

Each patient underwent CCTA and CT-FFR assessment without encountering any complications. Notably, both at discharge and six months post-TAVR, there was a significant improvement observed in the New York Heart Association (NYHA) functional classification, left ventricular fractional shortening, and ejection fraction compared to pre-operative levels. The CT-FFR for left anterior descending artery (LAD), left anterior descending artery (LCX), and right coronary artery (RCA) had no obvious change at discharge compared to pre-operation (0.92 ± 0.05 *vs.* 0.93 ± 0.05, *p* = 0.109; 0.96 ± 0.03 *vs.* 0.95 ± 0.03, *p* = 0.523; 0.97 ± 0.04 *vs.* 0.97 ± 0.03, *p* = 0.533; respectively). Furthermore, TAVR did not exert a significant impact on plaque burden during the perioperative period.

**Conclusions:**

Our report suggested that TAVR did not significantly affect coronary CT-FFR measurements and plaque characteristics in the perioperative period, and furthermore, the patients’ cardiac function showed gradual improvement in the short-term following discharge.

## 1. Introduction

Transcatheter aortic valve replacement (TAVR) has emerged as the preferred treatment for elderly patients with severe aortic valve stenosis due to its superior safety outcomes and reduced mortality rates compared to surgical intervention [1]. Computed tomography-derived fractional flow reserve (CT-FFR) has been incorporated into clinical guidelines as it effectively combines anatomical and functional ­information obtained from coronary computed tomography angiography (CCTA), offering a highly reliable diagnostic tool [2–10].

The safety, feasibility, and accuracy of CT-FFR in patients with severe aortic stenosis have resulted in its adoption in clinical practice and its potential for future research in functional coronary evaluation before TAVR [2–6,8–10]. The utilization of modern CT technology and an electrocardiogram (ECG)-gated retrospective technique has enabled the assessment of physiological function of coronary arteries through CT-FFR, offering valuable insights for TAVR planning in a substantial number of patients with severe aortic valve disease [5,6,8,9]. This diagnostic tool can effectively identify coronary artery stenoses and aid in determining the necessity of invasive coronary angiography for further evaluation [3–6,8,9].

However, the effect of transcatheter aortic valve replacement (TAVR) on changes of CT-FFR values was controversial [11,12]. Consequently, we formulated and executed an observational retrospective study with the aim of evaluating the clinical safety and feasibility of CT-FFR in this patient population. Specifically, we sought to identify the impact of TAVR on changes of CT-FFR values, plaque characteristics, and the associated clinical impact.

## 2. Patients and methods

### 2.1. Study design and participants

In this single-center observational study, 39 consecutive patients with severe aortic valve disease underwent TAVR between August 2019 and April 2023 with CCTA preoperatively and postoperatively. Exclusion criteria included prior stent implantation or coronary bypass surgery; contraindications to beta blockers, nitrates, or adenosine; suspicion of acute coronary syndrome; myocardial infarction within the last 3 months; and clinically significant arrhythmia. The flowchart of the patients included is presented in Supplementary Figure 1. According to the Declaration of Helsinki, the study was approved by the Institutional Ethics Committee of Fujian Medical University Union Hospital (approval number: 20230087). All subjects provided written informed consent.

### 2.2. Basic data collection

The research staff collected data on various clinical characteristics of the participants, including age, sex, body mass index, blood pressure, symptoms, lifestyle factors, such as smoking and alcohol habits, medical history encompassing hypertension, diabetes mellitus, ischemic stroke, chronic kidney diseases, atrial fibrillation, and premature ventricular contractions, as well as medication usage. For the purpose of this study, a current smoker was defined as an individual who had consistently smoked at least one cigarette per day for duration of six months or longer. A current drinker is someone who has consumed alcohol at least one time per week during the past year. The diagnosis of atrial fibrillation and premature ventricular contractions was made based on history and resting supine synchrony ECG.

Blood samples were collected using venipuncture after a minimum of a 12-h overnight fast. The blood samples were then analyzed for various biochemistries including hemoglobin, N-terminal pro brain natriuretic peptide (NT-proBNP), total cholesterol, total triglyceride, high-density lipoprotein cholesterol (HDL-C), low-density lipoprotein cholesterol (LDL-C), serum uric acid, and creatinine. These analyses were performed using automatic biochemical analyzers at the clinical laboratory of the Fujian Medical University Union Hospital. The glomerular filtration rate (eGFR) was estimated using the Chronic Kidney Disease Epidemiology Collaboration (CKD-EPI) equation.

### 2.3. TAVR procedures

Transcatheter aortic valve implantation was performed at digital subtraction angiography (Artis Zee ceiling, Simens, German or NeuAngio 30C Neusoft Medical Systems Co. Ltd., China) in accordance with the previously described method [1]. Throughout the study duration, the majority of valves utilized were second-generation self-expanding valves featuring an outer skirt and a supra-annular design. The domestic TaurusElite-AV valve (PEJIA MEDICAL) employed a bovine pericardial valve and was delivered *via* a 20-F sheath delivery system. In instances where deemed necessary, aortic valve pre-dilation or post-dilation was performed while employing rapid ventricular pacing. Intraoperative real-time catheter measurements were taken to ascertain the transvalvular peak pressure gradient.

### 2.4. CCTA acquisition

All CCTA image acquisitions were performed with a third-generation dual source 256-slice CT scanner (Somatom Force, Siemens) and included a prospectively ECG-triggered non-enhanced scan for calcium scoring. CCTA was conducted concurrently with CT for pre- and post-TAVR planning. A prospective ECG-gated helical scan of the heart was performed following the administration of 60 mL iopromide at a rate of 5 mL/s. Subsequently, coronary artery motion (including path and velocity) was examined and modeled in the frequency domain using SnapShot Freeze technology to efficiently reduce the reconstruction time window and obtain well-defined images of coronary artery anatomy [5,10]. The segmented coronary arteries were then visually and semi-automatically assessed for the severity of stenosis [2,3,6,10]. Furthermore, the determination of plaque characteristics (fibrous, calcified, lipid plaque, mixed) relied on the adaptive threshold method and the deep machine learning (ML) principle (medium membrane segmentation model) for quantitative analysis [7,13].

### 2.5. CT-FFR acquisition

**Figure 1.**
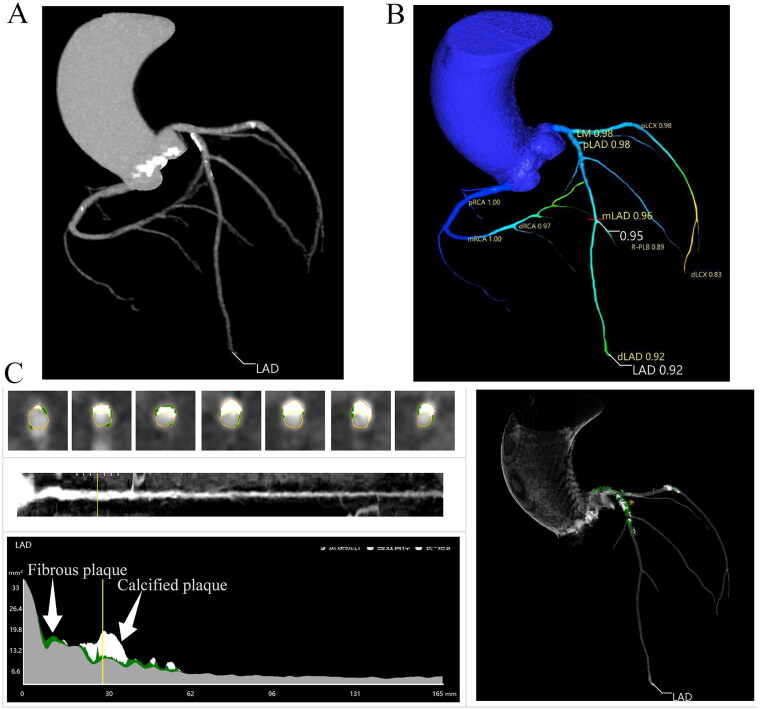
Representative images from oblique reformatted coronary CT angiography in a 77-year-old man with severe aortic stenosis. (A) Coronary CT angiography without obstructive coronary artery disease. (B) The final CT angiography–derived fractional flow reserve (FFR) results in major epicardial arteries. (C) The plaque characteristics analysis in LAD. CT: computerized tomography; LAD: left anterior descending artery; LCX: left circumflex artery; RCA: right coronary artery.

The calculation of CT-FFR was carried out by an independent core laboratory (Pulse Medical Imaging Technology, Shanghai, China) that was unaware of patient characteristics and clinical outcomes [14]. In this investigation, CT-FFR was derived from CCTA data sets using a previously described ML algorithm [6,9,15]. The ML-based CT-FFR algorithm was implemented on a dedicated workstation cFFR version 3.2 (Siemens; not commercially available) and trained using a sample of 12,000 synthetically generated coronary anatomies with randomly placed coronary stenoses, along with the results of computational fluid dynamics (CFD)–based CT-FFR [8–10,16]. On-site machine learning CT-FFRs have demonstrated faster processing times and have been validated against both CFD-based and invasive FFRs [6]. CT-FFR calculations were performed throughout the entire coronary tree, including all major epicardial arteries and side branches with a diameter of 2 mm or greater. CT-FFR was computed across the complete color-coded three-dimensional mesh of the coronary tree (Figure 1). The lowest CT-FFR value was utilized for per-patient analysis. The clinicians were unaware of the CT-FFR results, thereby ensuring that the clinical management was not influenced by the CT-FFR findings.

### 2.6. Clinical evaluation and follow-up

The Euro-SCORE II was utilized for preoperative risk assessment of TAVR in this study [17]. The use of the New York Heart Association (NYHA) functional classification was recommended to assess the severity of heart failure in patients. Patients were divided into four subgroups: no symptomatic patients (NYHA I), mildly symptomatic (NYHA II), moderately symptomatic (NYHA III), and severely symptomatic (NYHA IV). Meanwhile, all patients underwent thorough transthoracic echocardiography before the procedure, at discharge, at one and six month post-TAVR. Transthoracic ultrasonography was performed with the patient in the left lateral decubitus position, and the probe was closely attached to the sternum and apical regions of the subject to obtain two-dimensional and color Doppler flow images of the left ventricular long and short axis, apical long axis, apical four-chamber, and two-chamber views. They recorded the following parameters: left ventricular end diastolic dimension, left ventricular fraction shortening and ejection fraction, transvalvular peak pressure gradient, mean transvalvular pressure gradient, and dortic valve orifice area. Aortic regurgitation was evaluated before and after the procedure using a comprehensive multiparameter approaches, and its severity was categorized as none, mild, moderate, or severe, with further classification as transvalvular or paravalvular [18]. The primary outcome measure was defined as all-cause mortality, excluding nonfatal events.

### 2.7. Statistical analysis

Continuous or categorical variables were presented as the mean ± standard deviation or frequencies (percentage), respectively. The McNemar test was employed for comparing categorical variables, while the paired samples test was used for continuous variables. Statistical analysis was conducted using SPSS (version 25, IBM). All *p*-values were two-tailed, and a significance level of *p* < 0.05 was deemed statistically significant.

## 3. Results

A total of 117 vessels among 39 participants (mean age: 72.82 ± 6.68 years; male, 56.41%) were subjected to analysis. Hypertension and diabetes mellitus were observed in 26 patients (66.67%) and 10 patients (25.64%), respectively. Heart failure with preserved ejection fraction, mildly reduced ejection fraction, and reduced ejection fraction were identified in 24 patients (61.54%), four patients (10.26%), and five patients (12.82%), respectively. The clinical characteristics of participants are summarized in Table 1.

**Table 1.** Clinical characteristics of participants.

Characteristics	Total
Demographics	
Age (years)	72.82 ± 6.68
Male, *n* (%)	22 (56.41%)
Body mass index (kg/m^2^)	37.06 ± 5.45
Systolic blood pressure (mmHg)	126.62 ± 17.10
Diastolic blood pressure (mmHg)	70.64 ± 11.89
Heart rate (times/min)	75.56 ± 9.94
Clinical manifestation, risk factors, and concomitant diseases	
Typical angina, *n* (%)	27 (69.23%)
Dyspnea, *n* (%)	33 (84.62%)
Dizzy, *n* (%)	4 (10.26%)
Syncope, *n* (%)	3 (7.69%)
Smoking status, *n* (%)	
Never	26 (66.67%)
Former smoker	7 (17.95%)
Current smoker	6 (15.39%)
Drinking status, *n* (%)	
Never	32 (82.05%)
Former drinker	1 (2.56%)
Current drinker	6 (15.39%)
Hypertension, *n* (%)	26 (66.67%)
Diabetes mellitus, *n* (%)	10 (25.64%)
Dyslipidemia, *n* (%)	7 (17.95%)
History of stroke, *n* (%)	3 (7.69%)
Chronic kidney diseases, *n* (%)	5 (12.82%)
NYHA heart function grading, *n* (%)	
I	1 (2.56%)
II	7 (17.95%)
III	26 (66.67%)
IV	5 (12.82%)
HFpEF, *n* (%)	24 (61.54%)
HFmrEF, *n* (%)	4 (10.26%)
HFrEF, *n* (%)	5 (12.82%)
Atrial fibrillation, *n* (%)	8 (20.51%)
Premature ventricular contractions, *n* (%)	6 (15.39%)
Baseline blood values and electrocardiogram parameters	
Hemoglobin (g/L)	122.33 ± 19.28
NT-proBNP (pg/ml)	5108.59 ± 7466.18
Total cholesterol (mmol/L)	4.00 ± 1.28
Total triglyceride (mmol/L)	1.16 ± 0.42
HDL-C (mmol/L)	1.09 ± 0.33
LDL-C (mmol/L)	2.53 ± 1.04
Serum uric acid (μmol/L)	455.45 ± 117.43
Serum creatinine (μmol/L)	101.61 ± 94.45
eGFR (ml/min/1.73 m^2^)	73.72 ± 26.68
QRS duration (ms)	98.26 ± 27.43
PR interval (ms)	171.09 ± 19.86
Medication usage	
Diuretics, *n* (%)	28 (71.80%)
Spirolactone, *n* (%)	23 (58.97%)
Calcium channel blockers, *n* (%)	12 (30.77%)
ACEIs/ARBs, *n* (%)	17 (43.59%)
Sacubactril valsartan tablets, *n* (%)	17 (43.59%)
β-receptor blockers, *n* (%)	17 (43.59%)
Sodium–glucose co-transporter 2, *n* (%)	2 (5.13%)
Antiplatelet agents, *n* (%)	35 (89.74%)
Nnticoagulant drugs, *n* (%)	18 (46.15%)

NYHA: New York Heart Association; HFpEF: heart failure with preserved ejection fraction; HFmrEF: heart failure with mildly reduced ejection fraction; HFrEF: heart failure with reduced ejection fraction; NT-proBNP: N-terminal pro brain natriuretic peptide; HDL-C: high-density lipoprotein cholesterol; LDL-C: low-density lipoprotein cholesterol; eGFR: estimated glomerular filtration rate; ACEIs: angiotensin-converting enzyme inhibitors; ARBs: angiotensin receptor inhibitors.

*Note:* The data are shown as the mean ± standard deviations or *n* (%).

In our analysis, a total of 38 patients (97.44%) were diagnosed with severe aortic stenosis, while four patients (10.26%) had severe aortic regurgitations, as indicated in Table 2. The average Euro-SCORE II was found to be 3.17 ± 2.64%. The surgical approach involving the right femoral artery was the most commonly chosen method, accounting for 97.44% of cases. The mean calcification volumes of the aortic valve area were measured to be 670.36 ± 570.47 mm^3^, as shown in Table 2. Additionally, the ratio of artificial valve diameter to aortic valve annulus diameter was determined to be 0.93 ± 0.14, as indicated in Table 2. A total of 30 patients (70.92%) underwent general anesthesia, while 31 patients (71.49%) underwent pre-balloon dilatation and 16 patients (40.03%) underwent post-balloon dilatation with rapid ventricular pacing, as indicated in Table 2. Intraoperative real-time catheter measurements revealed that the preoperative and postoperative transvalvular peak pressure gradients were 75.22 ± 31.97 and 8.78 ± 6.59 mmHg, respectively. The observed change in transvalvular peak pressure ­gradient before and after the procedure was 66.44 ± 32.19 mmHg, as shown in Table 2.

**Table 2.** Valve implantation information of participants.

Characteristics	Total
Severe aortic stenosis, *n* (%)	38 (97.44%)
Aortic regurgitation, *n* (%)	
Mild	3 (7.69%)
Moderate	7 (17.95%)
Severe	4 (10.26%)
Euro-SCORE II scoring system	3.17 ± 2.64%
Main surgical approach, *n* (%)	
Right femoral artery	38 (97.44%)
Right common carotid artery	1 (2.56%)
Anesthesia method, *n* (%)	
General anesthesia	30 (70.92%)
Sedation induction and local anesthesia	9 (23.08%)
Calcification volumes of aortic valve area (mm^3^)	670.36 ± 570.47
Aotic valve annulus diameter (mm)	25.24 ± 4.18
Valve type, *n* (%)	
#FG12000-AV23	13 (33.33%)
#FG12000-AV26	20 (51.28%)
#FG12000-AV29	5 (12.82%)
#FG12000-AV31	1 (2.56%)
Days of cardiac CT scan before TAVI	4.85 ± 1.25
Days of cardiac CT scan after TAVI	7.31 ± 1.59
Artificial valve diameter/aotic valve annulus diameter	0.93 ± 0.14
ECMO assisted, *n* (%)	1 (2.56%)
Coronary protection, *n* (%)	1 (2.56%)
Pre-balloon dilatation, *n* (%)	31 (71.49%)
Post-balloon dilatation, *n* (%)	16 (40.03%)
Perivalvular leakage, *n* (%)	
Slight	2 (5.13%)
Mild	20 (53.85%)
Mild to moderate	5 (12.82%)
Preoperative transvalvular peak pressure gradient (mmHg)	75.22 ± 31.97
Postoperative transvalvular peak pressure gradient (mmHg)	8.78 ± 6.59
Changes of transvalvular peak pressure gradient before and after the procedure (mmHg)	66.44 ± 32.19

ECMO: extracorporeal membrane oxygenation.

*Note:* The data are shown as the mean ± standard deviations or *n* (%).

The left ventricular fraction shortening (LVFS) and ejection fraction (LVEF) showed significant improvement at the six-month follow-up after transcatheter aortic valve replacement (TAVR) compared to pre-operation (32.05 ± 10.87 *vs.* 37.84 ± 5.95%, *p* = 0.004; 58.30 ± 15.78 *vs.* 67.40 ± 7.48%, *p* = 0.002; respectively; see Table 3). Additionally, the left ventricular end diastolic dimension demonstrated a significant decrease at discharge and at the six-month follow-up after TAVR compared to pre-operation (52.63 ± 8.20 *vs.* 47.92 ± 8.20 mm, *p* < 0.001; 52.63 ± 8.20 *vs.* 49.32 ± 6.93 mm, *p* = 0.012; respectively).

**Table 3.** Echocardiogram indices and heart function grading at baseline and follow-up.

Characteristics	Pre-operation	At discharge	One month after discharge	Six months after discharge	*p*1-Value	*p*2-Value	*p*3-Value
Left ventricular end diastolic dimension (mm)	52.63 ± 8.20	47.92 ± 8.20	49.33 ± 6.93	49.32 ± 6.93	<0.001	0.012	0.012
Left ventricular fraction shortening (%)	32.05 ± 10.87	31.81 ± 10.03	34.37 ± 7.30	37.84 ± 5.95	0.874	0.135	0.004
Left ventricular ejection fraction (%)	58.30 ± 15.78	58.73 ± 14.78	63.09 ± 10.51	67.40 ± 7.48	0.844	0.040	0.002
Transvalvular peak pressure gradient (mmHg)	89.03 ± 29.17	25.18 ± 10.53	26.29 ± 11.29	25.13 ± 12.29	<0.001	<0.001	<0.001
Mean transvalvular pressure gradient (mmHg)	55.03 ± 18.04	14.05 ± 5.86	14.68 ± 6.66	14.31 ± 7.41	<0.001	<0.001	<0.001
Aortic valve orifice area (cm^2^)	0.71 ± 0.41	1.77 ± 0.58	1.67 ± 0.74	1.87 ± 0.80	<0.001	<0.001	<0.001
NYHA heart function grading	2.90 ± 0.64	2.62 ± 0.85	2.10 ± 0.45	1.21 ± 0.41	0.003	<0.001	<0.001

*p*1-value represented pre-operation *vs.* at discharge; *p*2-value represented pre-operation *vs.* one month after discharge; *p*3-value represented pre-operation *vs.* six months after discharge.

*Note:* The data are shown as the mean ± standard deviations.

The echocardiogram measurements revealed that the preoperative transvalvular peak and mean pressure gradients were 89.03 ± 29.17 and 55.03 ± 18.04 mmHg, respectively. Similarly, the transvalvular peak and mean pressure gradients at discharge were measured to be 25.18 ± 10.53 and 14.05 ± 5.86 mmHg, respectively. Furthermore, the echocardiogram measurements indicated that the aortic valve area at pre-operation and at discharge were 0.71 ± 0.41 and 1.77 ± 0.58 cm^2^, respectively.

The NYHA heart function grading showed a significant improvement at discharge and at the six-month follow-up after TAVR, compared to the pre-operation state (2.90 ± 0.64 *vs.* 2.62 ± 0.85, *p* = 0.003; 2.90 ± 0.64 *vs.* 1.21 ± 0.41, *p* < 0.001 = 0.012; respectively). There was only one reported case of severe pneumonia resulting in death on the 19th day post-surgery. None of the patients experienced adverse cardiac outcomes, including cardiac mortality, nonfatal myocardial infarction, or any revascularization, during the six-month follow-up after TAVR.

All patients underwent CTA and CT-FFR assessment without any complications. Upon conducting a per vessel analysis, it was observed that the CT-FFR values for the left anterior descending artery (LAD), left circumflex artery (LCX), and right coronary artery (RCA) showed no significant alteration at the time of discharge in comparison to the pre-operative measurements (0.92 ± 0.05 *vs.* 0.93 ± 0.05, *p* = 0.109; 0.96 ± 0.03 *vs.* 0.95 ± 0.03, *p* = 0.523; 0.97 ± 0.04 *vs.* 0.97 ± 0.03, *p* = 0.533; respectively, Table 4).

**Table 4.** The numeric value of CT-derived fractional flow reserve and plaque characteristics of coronary artery.

Characteristics	Pre-operation	At discharge	*p*-Value
CT-FFR for LAD	0.92 ± 0.05	0.93 ± 0.05	0.109
CT-FFR for LCX	0.96 ± 0.03	0.95 ± 0.03	0.523
CT-FFR for RCA	0.97 ± 0.04	0.97 ± 0.03	0.533
Plaque characteristics			
LAD			
Total plaque volume (mm^3^)	102.76 ± 131.66	96.63 ± 136.06	0.717
Plaque burden (%)	37.03 ± 25.70	37.08 ± 27.74	0.167
Calcification plaque volume (mm^3^)	36.84 ± 71.26	47.17 ± 87.40	0.059
Minimum lumen diameter (mm)	3.09 ± 1.27	3.21 ± 1.29	0.697
LCX			
Total plaque volume (mm^3^)	17.94 ± 30.47	11.60 ± 17.06	0.160
Plaque burden (%)	22.26 ± 20.93	18.92 ± 20.16	0.257
Calcification plaque volume (mm^3^)	7.44 ± 17.79	3.53 ± 8.29	0.073
Minimum lumen diameter (mm)	3.14 ± 0.97	3.34 ± 1.35	0.427
RCA			
Total plaque volume (mm^3^)	37.57 ± 62.52	48.99 ± 89.09	0.324
Plaque burden (%)	24.25 ± 23.40	27.56 ± 29.69	0.379
Calcification plaque volume (mm^3^)	13.65 ± 27.66	21.23 ± 44.34	0.153
Minimum lumen diameter (mm)	4.33 ± 6.66	2.59 ± 1.16	0.211
Roots of area moderate stenosis (≥50%), *n* (%)	20 (17.09%)	22 (18.80%)	0.623
Roots of area severe stenosis (≥70%), *n* (%)	1 (0.86%)	1 (0.86%)	1.000

CT-FFR: computerized tomography-derived fractional flow reserve; LAD: left anterior descending artery; LCX: left circumflex artery; RCA: right coronary artery.

*Note:* The data are shown as the mean ± standard deviations or *n* (%).

There was no significant alteration in the total volume or burden of plaque in the LAD, LCX, and RCA at the time of discharge compared to pre-operation, as observed during plaque characteristics analysis (102.76 ± 131.66 *vs.* 96.63 ± 136.06 mm^3^, *p* = 0.717 or 37.03 ± 25.70 *vs.* 37.08 ± 27.74%, *p* = 0.167; 17.94 ± 30.47 *vs.* 11.60 ± 17.06 mm^3^, *p* = 0.160 or 22.26 ± 20.93 *vs.* 18.92 ± 20.16%, *p* = 0.257; 37.57 ± 62.52 *vs.* 48.99 ±89.09 mm^3^, *p* = 0.324 or 24.25 ± 23.40 *vs.* 27.56 ± 29.69%, *p* = 0.379; respectively, Table 4). Before the operation, 13 patients (33.33%) exhibited moderate stenosis (50–69%) in the lumen area of twenty coronary arteries, as determined by CCTA. Following the operation, 17 patients (43.59%) displayed moderate stenosis (50–69%) in the lumen area of twenty-two coronary arteries. Notably, only one patient exhibited severe stenosis (≥70%) in a coronary vessel both before and after the operation.

## 4. Discussion

Coronary evaluation by CTA before TAVI is not yet standard practice. The findings of our study indicated the safety and feasibility of coronary CTA and CT-FFR in patients who had undergone TAVR for severe aortic valve disease. Notably, we observed no significant disparity in CT-FFR values in the main epicardial blood vessels at discharge compared to pre-operative values, suggesting that TAVR did not have a substantial impact on plaque burden during the perioperative period. Moreover, we observed a gradual improvement in short-term cardiac function in these patients following discharge. As a result of our study, we made significant progress in assessing coronary physiology noninvasively in such cohorts.

In the context of patients undergone TAVR, invasive coronary angiography (ICA) has traditionally been employed for revascularization determinations and procedural risk assessment. However, it lacks the capability to offer insights into the functional consequences of coronary stenosis, which could potentially aid in guiding revascularization decisions before TAVR [19]. Consequently, the FFR has emerged as a clinically validated parameter upon which revascularization decisions are predicated [20]. In the population of patients diagnosed with aortic stenosis, those who received revascularization guided by FFR exhibited a higher rate of successful major adverse cardiovascular events-free survival compared to those who solely relied on angiographic guidance [19]. Given the elevated surgical risks associated with invasive coronary angiography (ICA) and FFR in this high-risk cohort, which frequently comprises elderly individuals with comorbidities, there exists a persistent requirement for a non-invasive approach to identify lesion-specific ischemia.

The CCTA is a widely employed noninvasive technique for diagnosing and treating individuals experiencing recent onset of chest pain. It proves particularly advantageous in evaluating patients with low to intermediate pre-test probabilities for coronary heart disease (CAD) owing to its remarkable negative predictive value. The CT-FFR, a clinically-applied modality, employs computational flow dynamics to simulate invasive FFR based on a standard CTA acquisition [19].

CT-FFR was found to be both technically feasible and safe for implementation during TAVR, without necessitating any additional scan time or modifications to the CT scan protocol. Previous studies have examined the application of coronary CT-FFR with enhanced imaging acquisition in individuals with severe aortic valve disease, specifically aortic stenosis [2–4,8–12]. Given its established accuracy in evaluating coronary artery stenoses, the utilization of computed CT-FFR for coronary clearance in TAVR procedures is increasingly prevalent [3]. The implementation of CCTA and CT-FFR in the preoperative assessment of CAD in patients undergoing TAVR evaluation has the potential to enhance patient outcomes and satisfaction by offering cost-effective, highly dependable, and precise evaluations of hemodynamic stenosis in the native coronary arteries [3]. Aquino et al. [6] found that CT-FFR was associated with major adverse cardiac events in candidates for TAVR. The addition of CT-FFR data to CCTA and clinical data improved their predictive power for major adverse cardiovascular events, but not for all-cause mortality [6]. In addition to improving diagnostic accuracy, CT-FFR possibly reduced the risks associated with ICA by reducing the need for additional testing [8,9].

The diagnostic efficacy of CT-FFR is generally diminished in individuals with severe aortic stenosis compared to those with stable CAD and no severe aortic stenosis [2,13]. In patients with severe aortic stenosis, the presence of pathophysiological alterations in coronary and microcirculatory flow may contribute to this observation [21]. The occurrence of valvular stenosis leads to structural, microcirculatory, and neurohumoral modifications, resulting in compromised coronary flow reserve and myocardial ischemia, even in the absence of significant coronary stenosis [21].

It was found that TAVR directly improved CT-FFR values in patients with compromised coronary flow due to the normalization of sympathetic nervous system hyperactivity [11]. However, patients with a history of CAD, particularly those with severely stenosed lesions in the LAD artery, were susceptible to a change in their CT-FFR from a negative value (>0.80) to a positive value (≤0.80) following TAVR [11]. A reliable way to assess coronary functions and the corresponding cutoff value for a clinically significant lesion in TAVR patients is desirable to guide patient management. Michiels et al. [12] found no confounding effect of left ventricular mass regression after TAVR or surgical aortic valve replacement on CT-FFR values. In accordance with our study’s outcomes, there was notable left ventricular remodeling observed six months after aortic stenosis treatment [12]. However, the CT-FFR values remained unchanged. Our findings indicated that TAVR did not have a substantial effect on coronary CT-FFR measurements or plaque accumulation during the perioperative period. It is worth noting that our study population had a limited number of cases with CAD, which might account for the varying impact of TAVR on CT-FFR value alterations across different study populations.

We also considered that the hemodynamic effects of the original aortic stenosis induced small changes in CT-FFR measurements. On the other hand, the normalization of sympathetic nervous system hyperactivity and hemodynamic improvement after the procedure offset the effect of the regional disturbances generated by the transcatheter heart valves on in CT-FFR values. There is a need for further studies comparing the performance of the different invasive and non-invasive coronary physiological indexes in this patient group. Subsequent prospective trials might provide further validation for the potential utility of routine CT-FFR measurements in assessing functional coronary parameters among patients with severe aortic valve disease undergoing TAVR.

This study presented several limitations. Firstly, there were inherent biases and limitations in this single-center study. The small sample size raised concerns about the generalizability of the findings to other regions and ethnic populations. Secondly, the relatively young mean age of 72.8 years in our cohort undergoing TAVR raises questions about the applicability of these findings to older patients. Additionally, the interval between the pre- and post-procedural CT-FFR measurements is too short to have a meaningful impact on coronary plaque burden. As such, the CT-FFR value would not be or with only very weakly affected by a change in plaque burden. Thus, it remains uncertain whether the results can be extrapolated to the older population indeed appropriate. Besides, this study focuses solely on one commercially available CT-FFR technique, leaving the validity of other invasive techniques unexplored. Finally, our study had limited power for the evaluation of cardiac death and myocardial infarction due to the small sample size.

## 5. Conclusions

Our findings demonstrated that patients with severe aortic valve disease who underwent TAVR were able to safely and successfully undergo coronary CTA and CT-FFR. Our report suggested that TAVR did not significantly affect coronary CT-FFR measurements and plaque characteristics in the perioperative period, and furthermore, the patients’ cardiac function showed gradual improvement in the short-term following discharge. The present preliminary data should serve as a basis for future research into the use of invasive FFRs during TAVR procedural planning.

## Supplementary Material

Supplemental Material

## Data Availability

The datasets used and/or analyzed during the present study are available from the corresponding author on reasonable request.
