# Supplementary material for: The impact of transcatheter aortic valve replacement on changes of coronary computed tomography-derived fractional flow reserve
Source: Ann Med. 2024 Oct 28;56(1):2420860. doi: 10.1080/07853890.2024.2420860 (PMC11520094; doi:10.1080/07853890.2024.2420860)
Supplement: Supplemental Material [file IANN_A_2420860_SM2264.docx]

**Supplementary figure**


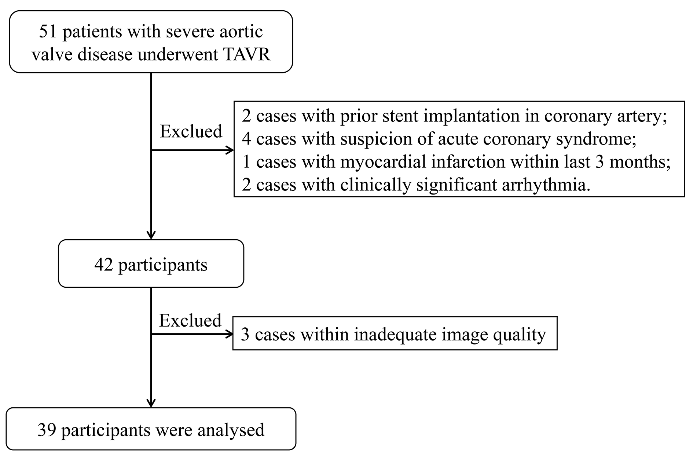


**Supplementary figure 1** Data flow chart of participants in our analysis. Abbreviations: TAVR, transcatheter aortic valve replacement.
